# First successful case of platinum‐based chemotherapy for neuroendocrine prostate cancer with *BRCA2* and *PTEN* alterations

**DOI:** 10.1002/iju5.12383

**Published:** 2021-10-20

**Authors:** Minami Omura, Takeo Kosaka, Eriko Aimono, Kohei Nakamura, Hiroshi Hongo, Shuji Mikami, Hiroshi Nishihara, Mototsugu Oya

**Affiliations:** ^1^ Department of Urology Keio University School of Medicine Tokyo Japan; ^2^ Genomics Unit Keio Cancer Center Keio University School of Medicine Tokyo Japan; ^3^ Division of Diagnostic Pathology Keio University Hospital Tokyo Japan

**Keywords:** *BRCA2* mutation, case report, DNA repair, prognosis, treatment outcome

## Abstract

**Introduction:**

Deoxyribonucleic acid repair gene mutations are now being studied in a variety of solid tumors, with the hope of predicting prognosis, pathogenesis, and treatment outcomes.

**Case presentation:**

We report the case of a Japanese patient with advanced castration‐resistant prostate cancer who exhibited a prominent response to platinum therapy and had coexisting *BRCA2* and *PTEN* mutations according to retrospective multigene panel analysis.

**Conclusion:**

Through a review of clinical outcomes and genetic/pathologic profiling, the presented case provides insights into future management strategies based on the tumor genetic status.

**Abbreviations & Acronyms**
AR: androgen receptor
CBDCA: carboplatin
CBZ: cabazitaxel
CRPC: castration‐resistant prostate cancer
eCN: estimated copy number
DNA: deoxyribonucleic acid
DOC: docetaxel
DSB: double‐strand break
LHRH: luteinizing hormone‐releasing hormone
NSE: neuron specific enolase
PSA: prostate‐specific antigen
TUR‐P: transurethral resection of the prostate
VP‐16: etoposide

**Keynote message**

We present a Japanese patient with CRPC and coexisting *BRCA2* and *PTEN* mutations who displayed a prominent response to platinum therapy. The patient exhibited atypically long survival despite high‐risk clinical and pathological features, and the relationships of disease progression and treatment response with the genetic status were explored. Our study provides insights into future management strategies based on the tumor genetic status.

## Introduction

DNA repair gene mutations have been studied in a variety of solid tumors and are now gathering attention in prostate cancer because of the possible effects on outcome and selection of treatment. Since June 2019 in Japan, a gene panel analysis system under the name of OncoGuide NCC Oncopanel System and FoundationOne has been listed for coverage under the national health insurance and has provided opportunities to review atypical clinical outcomes in patients with cancer. In this report, we presented a case of long‐surviving CRPC with a prominent response to initial platinum therapy in a patient carrying a rare combination of somatic *BRCA2* and *PTEN* mutations.

## Case presentation

**Fig. 1:**
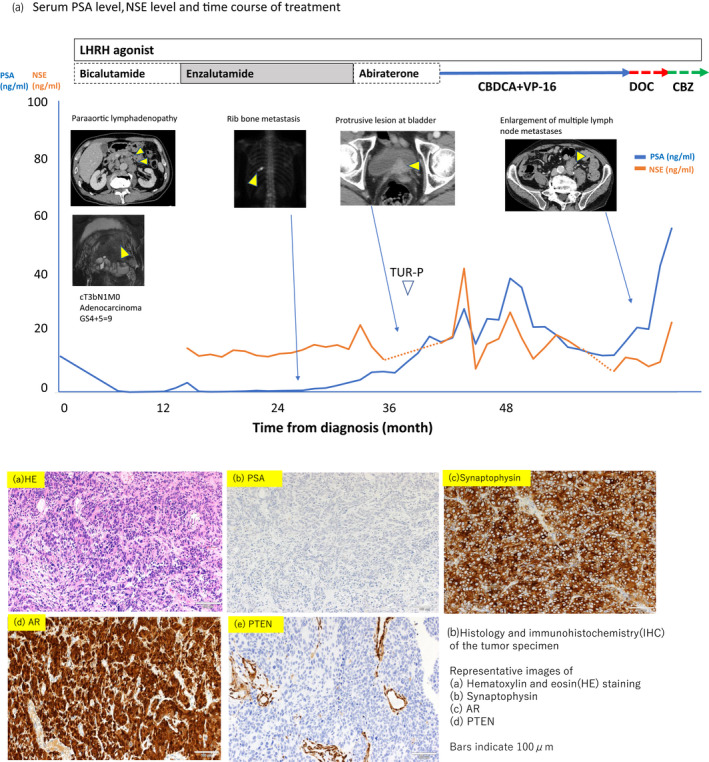
(a) Serum PSA level, NSE level and treatment time course of the study patient computed tomography and magnetic resonance imaging show response to therapy. (a) Low power view of tumor tissue stained with hematoxylin and eosin. Tissue immunostaining to detect (b) PSA, (c) synaptophysin, (d) AR, and (e) PTEN. The scale bar indicates 100 μm.

A 67‐year‐old man presented with an elevated PSA level (7.08 ng/mL) and signs of prostate enlargement on ultrasound during follow‐up of chronic hepatitis. Transrectal prostate needle biopsy revealed a pathologic diagnosis of adenocarcinoma with Gleason score of 4 + 5 = 9 in 9 of 12 specimens. Based on post‐biopsy images, the patient was staged as cT3bN1M1a with seminal vesicle involvement and lymphadenopathy within the left obturator fossa, paraaortic, supraclavicular, and mediastinal regions. Subsequently, he received combined androgen blockade therapy. After 27 months, a new metastasis to the rib was detected on bone scintigraphy, although his PSA level was controlled at 0.86 ng/mL (Fig. 1). As his PSA level reached 7.28 ng/ mL, based on a diagnosis of CRPC, the treatment was altered to abiraterone.

During abiraterone treatment, the patient’s PSA level increased accompanying elevation of neuron‐specific enolase and urination disorder secondary to obstruction. He underwent TUR, and the specimen displayed adenocarcinoma (Gleason score 5 + 5 = 10) with synaptophysin‐positive cells. Thus, he was diagnosed as adenocarcinoma with neuroendocrine differentiation (Fig. 1). Subsequent immunohistochemistry of his initial biopsy specimen revealed minor portions of synaptophysin‐ and chromogranin A‐positive cells.

Considering these results, platinum‐based treatment in combination with etoposide was selected (Fig. 1). After 11 courses, his serum PSA level decreased and his lymph nodes regressed. Owing to this drastic response, treatment was continued for 17 months, at which point his PSA level had increased to 22.53 ng/mL and lymphadenopathy recurred. The treatment was changed to DOC and then to CBZ because of PSA elevation, but the patient died before the approval and availability of olaparib.

After the approval of FoundationOne CDx, we performed next‐generation sequencing using his TUR specimen while the patient was still undergoing platinum‐based chemotherapy (Appendix S1; Table S1). All gene alterations are provided in Table S2. There were large deletions in *BRCA2* (exons 1–11) and *PTEN* (exons 2–3) accompanying biallelic loss (eCN was calculated as 0). *TP53* somatic frameshift mutation (p.A86fs*55) and *FANCA* somatic frameshift mutation (p.S849fs*40) were detected as a pathogenic variant in the tumor. Furthermore, AR gene amplification revealed an eCN of 10. Several other gene mutations were detected as a variant of unknown significance. The tumor mutation burden in the sample was 8.8 single nucleotide variants/Mbp.

Sequencing and immunohistochemistry were performed for both the tumor and intact regions, revealing large deletions in *BRCA2* and *PTEN* only in the tumor. The normal copy numbers of *BRCA2* and *PTEN* in normal prostatic tissue implied that these mutations were somatic events. The tumor cells showed reduced staining for PTEN, suggesting loss of expression at the protein level (Fig. 1).

## Discussion and conclusions

*BRCA2* is a tumor suppressor gene known for its role in the repair of DNA DSBs. Loss of *BRCA2* function leads to the failure of homologous recombination, making cells vulnerable to damage.^1^ Among patients with prostate cancer, up to 16.3% are reported to carry either germline or somatic *BRCA2* mutations.^2^ Germline *BRCA2* mutation is more common in metastatic hormone‐naïve prostate cancer than in localized disease (5.35% *vs* 0.87%).^3^ Moreover, patients with early‐onset prostate cancer have a relatively high rate of *BRCA2* mutation.^4^, ^5^

In ovarian and breast cancers, the presence of *BRCA2* mutation was mentioned to confer possible sensitivity to platinum agents and poly‐ADP ribose polymerase inhibitors.^6^, ^7^, ^8^ These agents are known to induce intrastrand and interstrand DNA damage, and similar responses are expected in prostate cancer.^9^, ^10^

Conversely, *PTEN* is a tumor suppressor gene known to inhibit signaling pathways related with tumor growth and migration.^11^ The loss of PTEN function was detected in up to 20% of primary prostate tumor samples obtained via radical prostatectomy,^12^, ^13^, ^14^, ^15^ and its frequency increased to as high as 50% in CRPC. Among patients treated with abiraterone, those with *PTEN* loss are known to have higher incidence of metastatic disease and significantly lower survival rates.^16^ Studies on treatment targeting the AKT signaling pathway are ongoing.^17^

As mentioned previously, *BRCA2* and *PTEN* mutations are commonly observed in advanced CRPC. However, to our surprise, we found that *BRCA2* and *PTEN* alterations were mutually exclusive in both primary^13^ and advanced^18^, ^19^ cancers (Table S3). Of the 333 primary and 150 advanced cases, only one case in each cohort displayed coexistent *BRCA2* and *PTEN* gene alterations (Table S3). The *BRCA2* and *PTEN* alterations were linked to features such as Gleason score 8 (4 + 4), T3a adenocarcinoma, and mortality within 4.8 months in the primary prostate cancer case (Table S4). Considering these findings, patients with coexistent *BRCA2* and *PTEN* alteration tend to have poor prognosis.

Clinically, our patient rapidly developed high‐grade metastatic disease, exhibiting a limited response to abiraterone. Pathologically, the patient was diagnosed with prostate cancer with neuroendocrine differentiation and displayed long survival after treatment with platinum agents. Through genome sequencing, we were able to discover the presence of somatic *BRCA2* and *PTEN* alterations in the background of the disease. The prominent response to platinum agents may be explained by loss of BRCA2 function and vulnerability to DSBs. However, the mechanism of the later resistance to platinum‐based chemotherapy remains an unanswered question. The frequency of somatic *BRCA2* and *PTEN* alterations in patients with neuroendocrine prostate cancer is unknown because of its rarity and the lack of available genomic analysis in the literature.

This patient unfortunately died before the approval of olaparib. If this treatment had been available, it would have likely been used in the first‐line setting in our patient. However, the outcome of this treatment would have been unpredictable. As gene panel analysis becomes accessible for daily practice, the accumulation of data is expected. As a first step, we reported a Japanese patient with neuroendocrine CRPC with coexistent somatic *BRCA2* and *PTEN* alterations displaying a prominent response to platinum therapy. Our retrospective report hopes to shed light on improving the survival of patients with high‐risk disease through adequate treatment selection based on early gene profiling.

## Funding

This work was supported in part by a Grant‐in‐Aid for Scientific Research (B: #20H03817 to TK) from the Ministry of Education, Culture, Sports, Science and Technology of Japan. The work was supported in part by a research grant to T. Kosaka from Keio Gijuku Fukuzawa Memorial Fund for the Advancement of Education and Research, Japan.

## Author contributions

Conception and design of the study: TK, MO, KN. Acquisition and analysis of data: TK, EA, HH, SM, KN, and HN. Drafting the manuscript and figures: TK, SM, HN, and MO. KN and HN were responsible for targeted next‐generation sequencing. Drafting the manuscript and figures: TK, SM, HN, and MO. All authors read and approved the final version of this manuscript.

## Conflict of interest

The authors declare no conflict of interest.

## Approval of the research protocol by an Institutional Reviewer Board

The study was approved by the by the Ethics Committee of Keio University Hospital (Approval numbers: 20160084 and 20180015).

## Informed consent

Consent to participate and for publication were acquired from the patient.

## Consent for publication

Consent for publication was acquired from the patient.

## Registry and the Registration No. of the study/trial

Not applicable.

## Supporting information


**Table S1.** 160 genes examined in the PleSSision‐Rapid test.

**Table S2.** Gene alterations in the study patient.

**Table S3.** Co‐occurrence of *BRCA2* and *PTEN* alterations analyzed using The Cancer Genome Atlas cohort data (a) and The SU2C−Prostate Cancer Foundation Prostate Dream Team cohort data (b).

**Table S4.** Characteristics of the BRCA2 / PTEN co‐alteration case in The Cancer Genome Atlas cohort.

**Appendix S1.** Materials and Methods for gene panel analysis

## References

[1] Prakash R , Zhang Y , Feng W *et al*. Homologous recombination and human health: the roles of BRCA1, BRCA2, and associated proteins. Cold Spring Harb. Perspect. Biol. 2015; 7: a016600. 10.1101/cshperspect.a016600. 25833843 PMC4382744

[2] Abida W , Armenia J , Gopalan A *et al*. Prospective genomic profiling of prostate cancer across disease states reveals germline and somatic alterations that may affect clinical decision making. JCO Precis. Oncol. 2017; 1: 1–16. 10.1200/PO.17.00029. PMC5558263 28825054

[3] Pritchard CC , Mateo J , Walsh MF *et al*. Inherited DNA‐repair gene mutations in men with metastatic prostate cancer. N. Engl. J. Med. 2016; 375: 443–53. 10.1056/NEJMoa1603144. 27433846 PMC4986616

[4] Mitra A , Fisher C , Foster CS *et al*. Prostate cancer in male BRCA1 and BRCA2 mutation carriers has a more aggressive phenotype. Br. J. Cancer 2008; 98: 502–7. 10.1038/sj.bjc.6604132. 18182994 PMC2361443

[5] Tryggvadóttir L , Vidarsdóttir L , Thorgeirsson T *et al*. Prostate cancer progression and survival in BRCA2 mutation carriers. J. Natl. Cancer Inst. 2007; 99: 929–35. 10.1093/jnci/djm005. 17565157

[6] Pennington KP , Walsh T , Harrell MI *et al*. Germline and somatic mutations in homologous recombination genes predict platinum response and survival in ovarian, fallopian tube, and peritoneal carcinomas. Clin. Cancer Res. 2014; 20: 764–75. 10.1158/1078-0432.CCR-13-2287. 24240112 PMC3944197

[7] Silver DP , Richardson AL , Eklund AC *et al*. Efficacy of neoadjuvant Cisplatin in triple‐negative breast cancer. J. Clin. Oncol. 2010; 28: 1145–53. 10.1200/JCO.2009.22.4725. 20100965 PMC2834466

[8] Papa A , Caruso D , Strudel M *et al*. Update on Poly‐ADP‐ribose polymerase inhibition for ovarian cancer treatment. J. Transl. Med. 2016; 14: 267. 10.1186/s12967-016-1027-1. 27634150 PMC5024442

[9] Mateo J , Carreira S , Sandhu S *et al*. DNA‐repair defects and olaparib in metastatic prostate cancer. N. Engl. J. Med. 2015; 373: 1697–708. 10.1056/NEJMoa1506859. 26510020 PMC5228595

[10] De Felice F , Tombolini V , Marampon F *et al*. Defective DNA repair mechanisms in prostate cancer: impact of olaparib. Drug Des. Devel. Ther. 2017; 11: 547–52. 10.2147/DDDT.S110264. PMC5338854 28280302

[11] Jamaspishvili T , Berman DM , Ross AE *et al*. Clinical implications of PTEN loss in prostate cancer. Nat. Rev. Urol. 2018; 15: 222–34. 10.1038/nrurol.2018.9. 29460925 PMC7472658

[12] Ahearn TU , Pettersson A , Ebot EM *et al*. A prospective investigation of PTEN loss and ERG expression in lethal prostate cancer. J. Natl. Cancer Inst. 2016; 108(2): djv346. 10.1093/jnci/djv346. 26615022 PMC4862436

[13] Cancer Genome Atlas Research Network . The molecular taxonomy of primary prostate cancer. Cell 2015; 163: 1011–25. 10.1016/j.cell.2015.10.025. 26544944 PMC4695400

[14] Suzuki H , Freije D , Nusskern DR *et al*. Interfocal heterogeneity of PTEN/MMAC1 gene alterations in multiple metastatic prostate cancer tissues. Cancer Res. 1998; 58: 204–9. 9443392

[15] Cairns P , Okami K , Halachmi S *et al*. Frequent inactivation of PTEN/MMAC1 in primary prostate cancer. Cancer Res. 1997; 57: 4997–5000. 9371490

[16] Ferraldeschi R , Nava Rodrigues D , Riisnaes R *et al*. PTEN protein loss and clinical outcome from castration‐resistant prostate cancer treated with abiraterone acetate. Eur. Urol. 2015; 67: 795–802. 10.1016/j.eururo.2014.10.027. 25454616 PMC4410287

[17] Kolinsky MP , Rescigno P , Bianchini D *et al*. A phase I dose‐escalation study of enzalutamide in combination with the AKT inhibitor AZD5363 (capivasertib) in patients with metastatic castration‐resistant prostate cancer. Ann. Oncol. 2020; 31: 619–25. 10.1016/j.annonc.2020.01.074. 32205016 PMC7217345

[18] Robinson D , Van Allen EM , Wu Y‐M *et al*. Integrative clinical genomics of advanced prostate cancer. Cell 2015; 161: 1215–28. 10.1016/j.cell.2015.05.001. 26000489 PMC4484602

[19] Hussain M , Daignault‐Newton S , Twardowski PW *et al*. Targeting androgen receptor and DNA repair in metastatic castration‐resistant prostate cancer: results from NCI 9012. J. Clin. Oncol. 2018; 36: 991–9. 10.1200/JCO.2017.75.7310. 29261439 PMC6075827

