# Supplementary figures and images for: First successful case of platinum‐based chemotherapy for neuroendocrine prostate cancer with BRCA2 and PTEN alterations
Source: IJU Case Rep. 2021 Oct 20;5(1):41–4. doi: 10.1002/iju5.12383 (PMC8720712; doi:10.1002/iju5.12383)

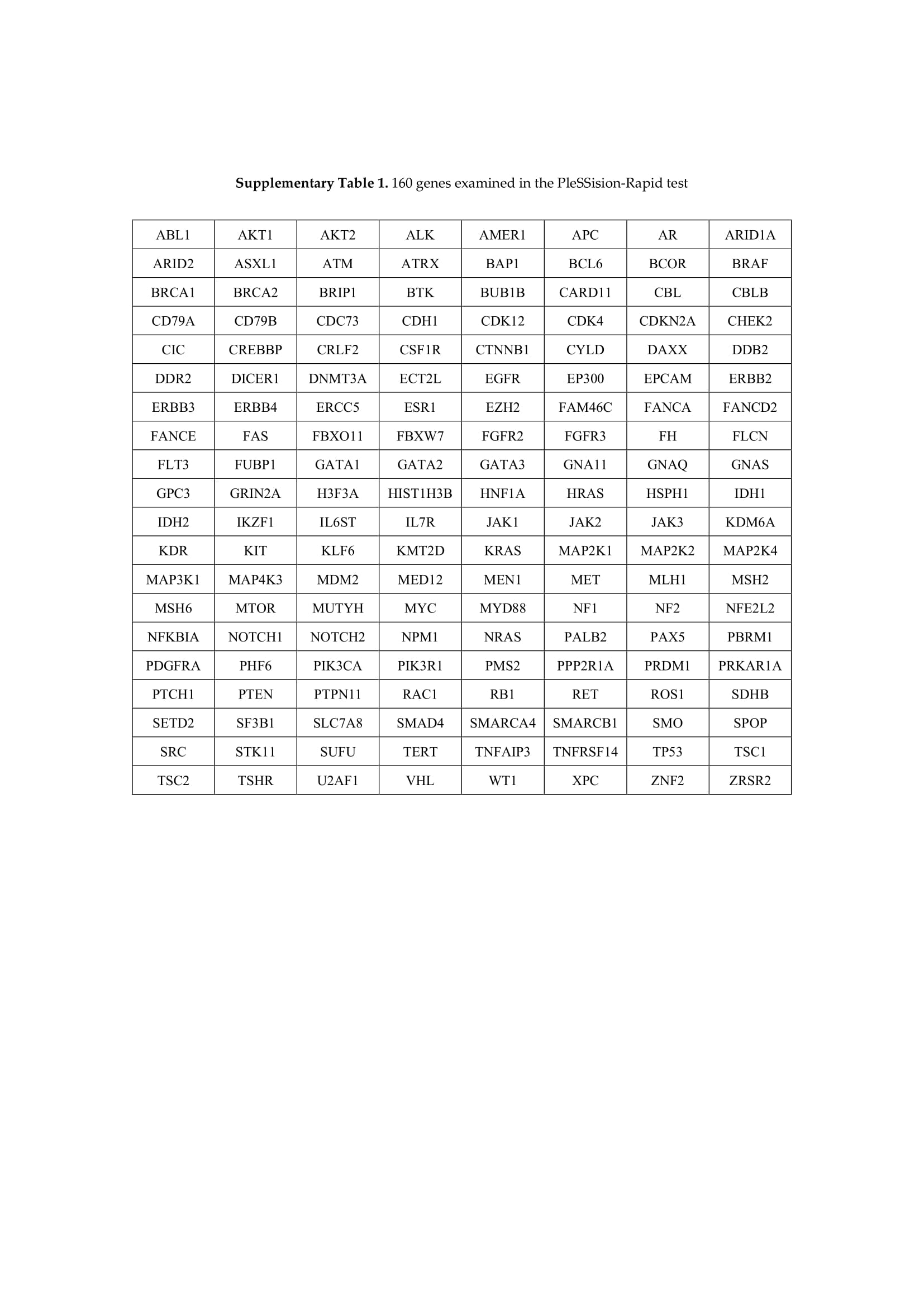

Supplement: Supplementary file 1 — Table S1. 160 genes examined in the PleSSision‐Rapid test. [file IJU5-5-41-s005.jpg]

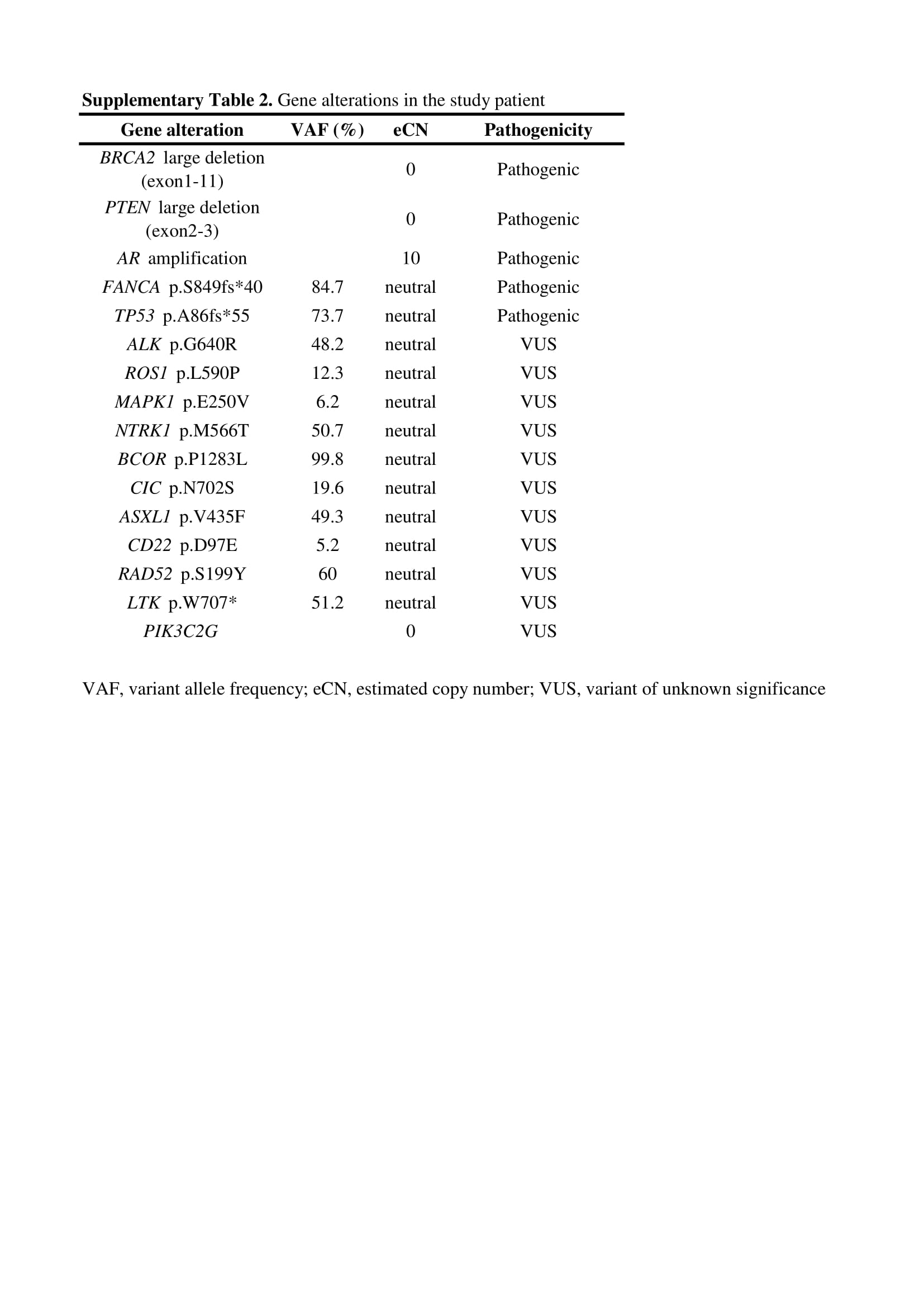

Supplement: Supplementary file 2 — Table S2. Gene alterations in the study patient. [file IJU5-5-41-s004.jpg]

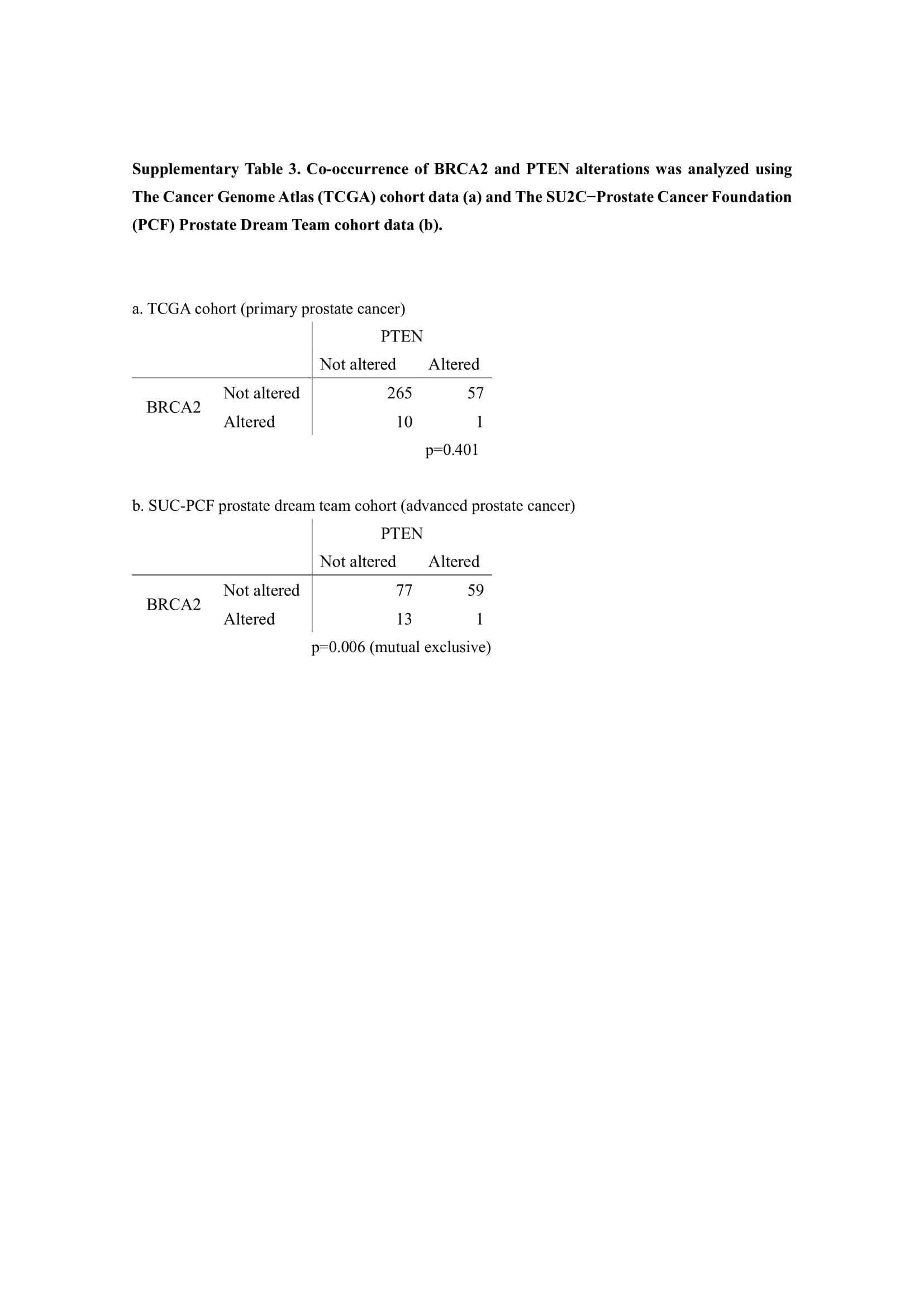

Supplement: Supplementary file 3 — Table S3. Co‐occurrence of BRCA2 and PTEN alterations analyzed using The Cancer Genome Atlas cohort data (a) and The SU2C−Prostate Cancer Foundation Prostate Dream Team cohort data (b). [file IJU5-5-41-s001.jpg]

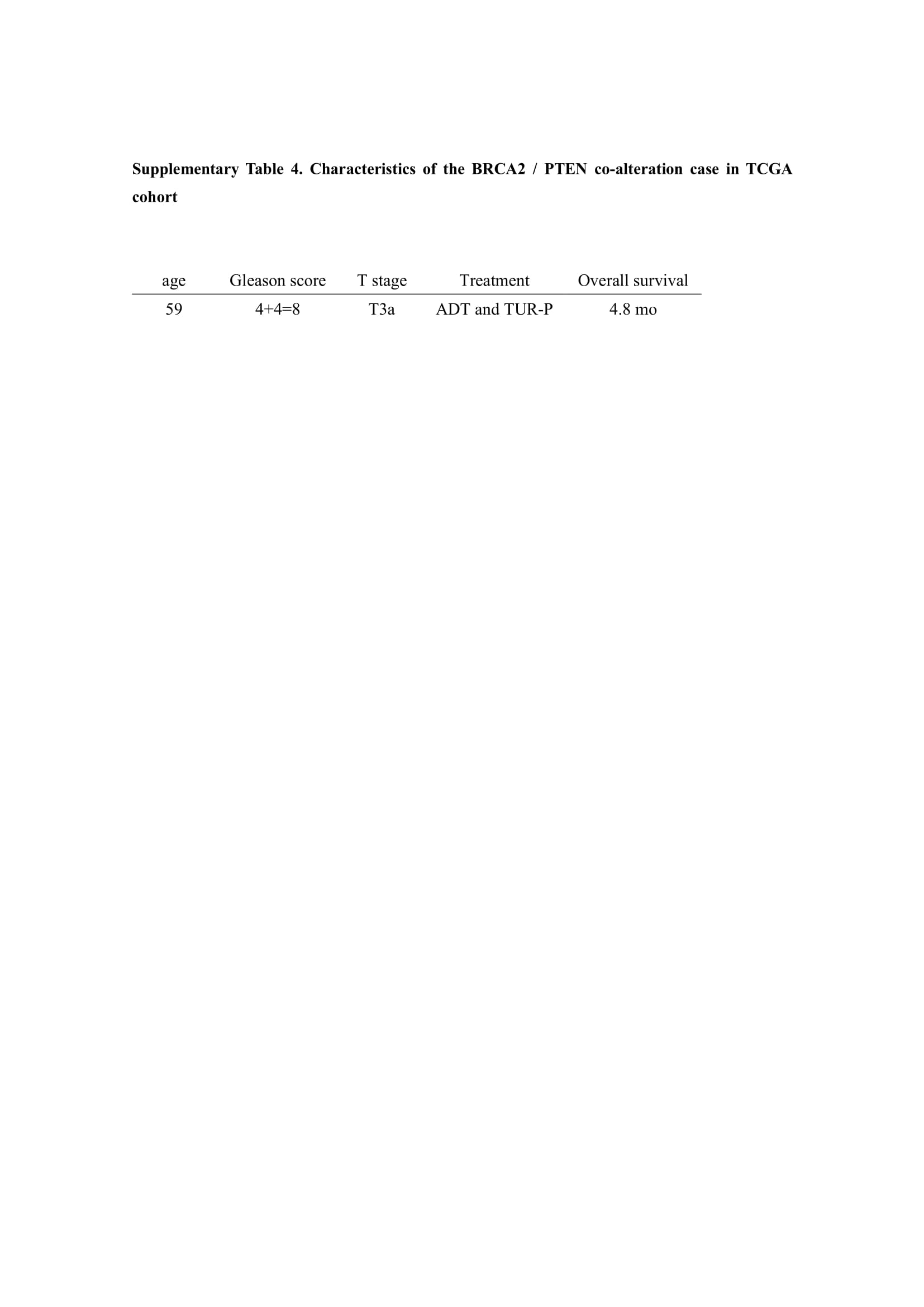

Supplement: Supplementary file 4 — Table S4. Characteristics of the BRCA2 / PTEN co‐alteration case in The Cancer Genome Atlas cohort. [file IJU5-5-41-s003.jpg]
