# Supplementary material for: First successful case of platinum‐based chemotherapy for neuroendocrine prostate cancer with BRCA2 and PTEN alterations
Source: IJU Case Rep. 2021 Oct 20;5(1):41–4. doi: 10.1002/iju5.12383 (PMC8720712; doi:10.1002/iju5.12383)
Supplement: Supplementary file 5 — Appendix S1. Materials and Methods for gene panel analysis [file IJU5-5-41-s002.docx]

Appendix

We investigated the frequency of mutations in 160 cancer-related genes in the prostate tumor specimen. Sections (10 µm) were dissected to provide > 20% tumor cells in the specimens and to minimize the presence of necrosis. Genomic testing was performed on a PleSSision-Rapid internal clinical sequencing apparatus, which is used for all genome sequencing-related analyses in our institution (Keio University Hospital). This apparatus was used to extract genomic DNA from tumor samples and peripheral blood mononuclear cells extracted from cancer patients, following the provision of consent to receive comprehensive genomic testing. This study was conducted in accordance with the Declaration of Helsinki and Title 45, U.S. Code of Federal Regulations, Part 46, Protection of Human Subjects, effective December 13, 2001.

DNA quality was checked by calculating the DNA integrity number (DIN) using an Agilent 2000 TapeStation (Agilent Technologies, Waldbronn, Germany) prior to conducting targeted amplicon exome sequencing of the 160 genes implicated in cancer using the Illumina MiSeq sequencing platform (Illumina, San Diego, CA). The 160 genes examined are listed in Supplementary Table S1. The smallest quantity of DNA had a DIN greater than 3.1. Sequencing data were entered into the GenomeJack bioinformatics pipeline (Mitsubishi Space Software, Tokyo, Japan) for analysis. Cancer-specific changes in somatic genes, including SNVs, insertions/deletions, and copy number variations were detected and used to determine the TMB.
